# Studies on the Regulation of Arginine Metabolism in Cyanobacteria Should Include Mixotrophic Conditions

**DOI:** 10.1128/mBio.01433-21

**Published:** 2021-06-22

**Authors:** Enrique Flores

**Affiliations:** a Instituto de Bioquímica Vegetal y Fotosíntesis, CSIC and Universidad de Sevilla, Seville, Spain; John Innes Centre; Yale School of Medicine

**Keywords:** arginine biosynthesis, arginine catabolism, mixotrophy, ornithine-ammonium cycle

## LETTER

**FIG 1:**
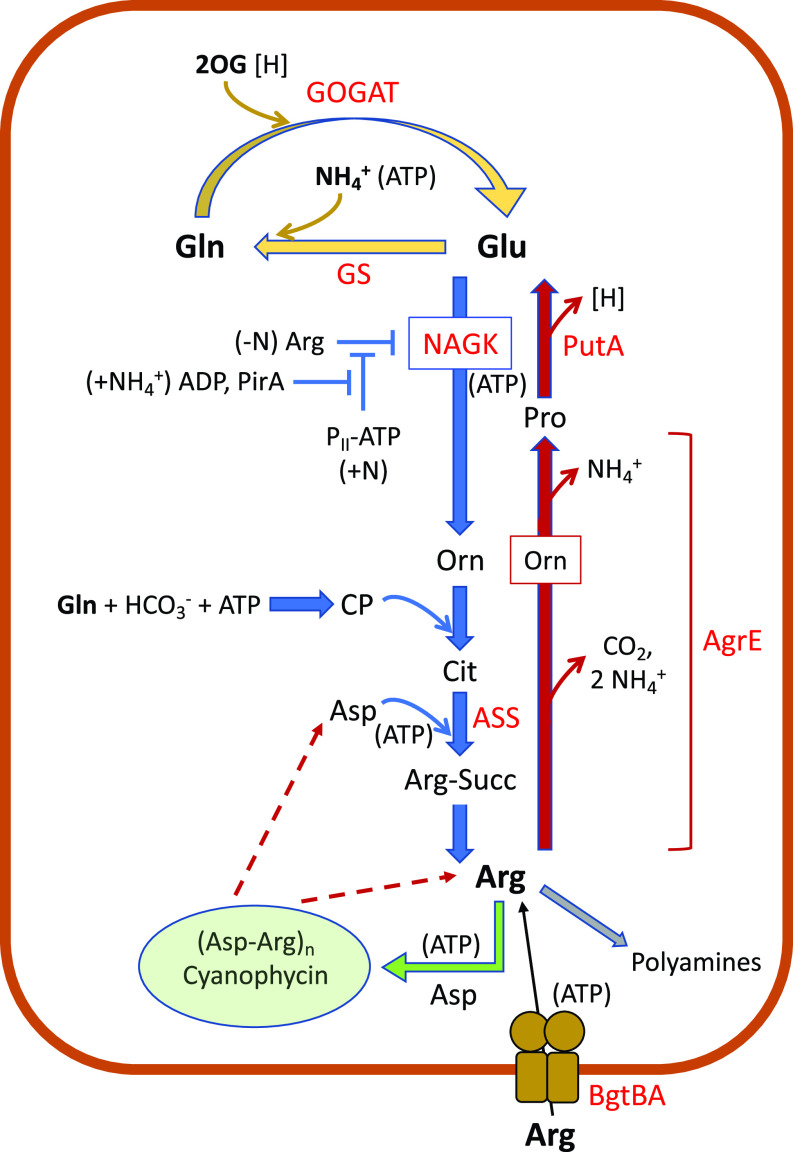
Schematic of arginine metabolism and some adjacent pathways in cyanobacteria. Arginine biosynthesis flux is shown in blue and arginine catabolism in garnet color. The upper part shows the GS/GOGAT pathway that produces the arginine precursor, glutamate, and the lower part shows arginine destinies other than protein synthesis, i.e., cyanophycin and polyamine synthesis. The regulatory network of NAGK is shown emphasizing functionality rather than molecular detail (i.e., NAGK-P_II_-ATP or P_II_-PirA complex formation is not indicated). For clarity, not all reactants, products, and stoichiometries are detailed, but places of energy input in the form of ATP are noted. Abbreviations: AgrE, arginine-guanidine-removing enzyme that carries sequential arginine dihydrolase and ornithine cyclodeaminase activities; Arg-Suc, argininosuccinate; ASS, argininosuccinate synthase; BgtBA, ABC transporter for basic amino acids and glutamine; Cit, citrulline; GS, glutamine synthetase; GOGAT, glutamine-2-oxoglutarate aminotransferase; [H], reducing power; 2OG, 2-oxoglutarate; Orn, ornithine; P_II_, *glnB* gene product; PirA, P_II_-interacting regulator of arginine synthesis; PutA, bifunctional proline oxidase that carries sequential proline dehydrogenase and glutamate-semialdehyde dehydrogenase activities.

In most bacteria ammonium assimilation takes place through the very efficient and ATP-consuming glutamine synthetase-glutamate synthase pathway, which produces two amino acids that are general distributors of nitrogen in cellular metabolism, glutamine and glutamate (1). A very direct use of these amino acids takes place in arginine biosynthesis, which starts with glutamate as precursor and in which another glutamate molecule, aspartate, and carbamoyl phosphate provide the three nitrogen atoms included in the guanidine group; carbamoyl phosphate is synthesized from glutamine, bicarbonate, and ATP (2). Given its high energetic demand, this pathway is usually subjected to feedback inhibition by arginine of one of its first enzymatic steps. In cyanobacteria, N-acetylglutamate kinase (NAGK) is inhibited by arginine, but under sufficient nitrogen, the C/N balance and energy status indicator P_II_ protein (*glnB* gene product) binds to NAGK, relieving its inhibition by arginine, with the effect of increasing the production of arginine by the biosynthetic pathway (3). Bolay et al. (4) now add another player to this regulatory system that they have identified in the unicellular cyanobacterium *Synechocystis*, the 51-amino-acid protein PirA. In response to ammonia upshifts, PirA binds to P_II_ in an ADP-dependent manner, preventing binding of P_II_ to NAGK and making this enzyme susceptible again to inhibition by arginine (Fig. 1).

The ammonia upshift has a general effect in *Synechocystis*, increasing the levels of many amino acids, notably glutamine, aspartate, arginine, and two arginine biosynthesis intermediates, ornithine and citrulline (4). Inactivation of *pirA* exacerbates the increase of aspartate, ornithine, and citrulline in response to the ammonia upshift, an effect that is counteracted by overexpression of *pirA*. Additionally, inactivation of *pirA* increases, and overexpression of *pirA* decreases, the levels of glutamate, proline, and some other amino acids, and overexpression of *pirA* decreases glutamine and arginine levels. These effects are consistent with a role of PirA in regulation of NAGK as shown by Bolay et al. (4), so that PirA appears to contribute to keep low levels of arginine biosynthesis intermediates downstream of NAGK as well as low levels of arginine and the arginine catabolism products proline and glutamate (see next paragraph). Nonetheless, the proposed mode of action of PirA, through ADP-dependent binding to P_II_, points to a possible more general effect of PirA in regulation of the C/N balance in cyanobacteria.

Bolay et al. (4) interpret their results as an effect of PirA controlling “flux into the ornithine-ammonia cycle.” This cycle (5) is a side activity of the arginine catabolism pathway and was defined before the whole activity of the key enzyme in cyanobacterial arginine catabolism, AgrE (arginine-guanidine-removing enzyme [6, 7]), was known. In the arginine catabolism pathway, AgrE (bifunctional arginine dihydrolase/ornithine cyclodeaminase) produces proline from arginine with ornithine as an intermediate, releasing one CO_2_ and three NH_4_^+^ molecules, and proline is further catabolized to glutamate by PutA (Fig. 1). It is possible that some ornithine is released from AgrE reentering the arginine biosynthesis pathway, hence the “ornithine-ammonia cycle” (5), described as a “urea cycle” previously, when the involvement of an enzyme with arginine dihydrolase activity was unknown (8). However, conditions influencing the balance between release of ornithine from AgrE and completion of the full AgrE activity (leading to proline) have not been investigated.

Many cyanobacteria produce cyanophycin, a copolymer of aspartate and arginine (multi-l-arginyl-poly[l-aspartic acid]) in which nitrogen can be stored under unbalanced growth with nonlimiting nitrogen (2). Significantly, in *Synechocystis*, a P_II_-dependent active NAGK is necessary to produce arginine that is accumulated in cyanophycin (9). Additionally, many cyanobacteria, including *Synechocystis*, express a high-affinity ABC transporter for basic amino acids (2, 10). This transporter allows *Synechocystis* cells, when supplied with micromolar concentrations of arginine, to accumulate arginine to about 10 mM (8). Because arginine biosynthesis and arginine catabolism might constitute a futile cycle in which much energy in the form of ATP is spent, it can be suggested that a main role of the regulation of the arginine biosynthesis pathway effected by feedback inhibition of NAGK is to avoid arginine biosynthesis when cyanophycin is being mobilized or when the cells encounter arginine in the environment. Indeed, cyanobacteria in general have an extensive capability for organic substrate transport (11), pointing to mixotrophy instead of simple inorganic ion assimilation/autotrophy as an important growth regime under natural conditions. Therefore, to fully understand the role of regulators such as PirA, further work on the physiology of arginine metabolism in cyanobacteria should address not only transitions between inorganic sources of nitrogen but also growth conditions in which arginine becomes available.

## References

[1] Herrero A, Flores E, Imperial J. 2019. Nitrogen assimilation in bacteria, p 280–300. *In* Schmidt TM (ed), Encyclopedia of microbiology, 4th ed, vol 3. Academic Press, Cambridge, MA.

[2] Flores E, Arévalo S, Burnat M. 2019. Cyanophycin and arginine metabolism in cyanobacteria. Algal Res 42:101577. doi:10.1016/j.algal.2019.101577.

[3] Forchhammer K, Selim KA. 2020. Carbon/nitrogen homeostasis control in cyanobacteria. FEMS Microbiol Rev 44:33–53. doi:10.1093/femsre/fuz025. 31617886 PMC8042125

[4] Bolay P, Rozbeh R, Muro-Pastor MI, Timm S, Hagemann M, Florencio FJ, Forchhammer K, Klähn S. 2021. The novel P_II_-interacting protein PirA controls flux into the cyanobacterial ornithine-ammonia cycle. mBio 12:e00229-21. doi:10.1128/mBio.00229-21. 33758091 PMC8092223

[5] Zhang H, Liu Y, Nie X, Liu L, Hua Q, Zhao GP, Yang C. 2018. The cyanobacterial ornithine-ammonia cycle involves an arginine dihydrolase. Nat Chem Biol 14:575–581. doi:10.1038/s41589-018-0038-z. 29632414

[6] Burnat M, Picossi S, Valladares A, Herrero A, Flores E. 2019. Catabolic pathway of arginine in *Anabaena* involves a novel bifunctional enzyme that produces proline from arginine. Mol Microbiol 111:883–897. doi:10.1111/mmi.14203. 30636068

[7] Lee H, Rhee S. 2020. Structural and mutational analyses of the bifunctional arginine dihydrolase and ornithine cyclodeaminase AgrE from the cyanobacterium *Anabaena*. J Biol Chem 295:5751–5760. doi:10.1074/jbc.RA120.012768. 32198136 PMC7186175

[8] Quintero MJ, Muro-Pastor AM, Herrero A, Flores E. 2000. Arginine catabolism in the cyanobacterium *Synechocystis* sp. strain PCC 6803 involves the urea cycle and arginase pathway. J Bacteriol 182:1008–1015. doi:10.1128/JB.182.4.1008-1015.2000. 10648527 PMC94377

[9] Maheswaran M, Ziegler K, Lockau W, Hagemann M, Forchhammer K. 2006. P_II_-regulated arginine synthesis controls accumulation of cyanophycin in *Synechocystis* sp. strain PCC 6803. J Bacteriol 188:2730–2734. doi:10.1128/JB.188.7.2730-2734.2006. 16547064 PMC1428389

[10] Quintero MJ, Montesinos ML, Herrero A, Flores E. 2001. Identification of genes encoding amino acid permeases by inactivation of selected ORFs from the *Synechocystis* genomic sequence. Genome Res 11:2034–2040. doi:10.1101/gr.196301. 11731493 PMC311220

[11] Stebegg R, Schmetterer G, Rompel A. 2019. Transport of organic substances through the cytoplasmic membrane of cyanobacteria. Phytochemistry 157:206–218. doi:10.1016/j.phytochem.2018.08.013. 30447471

